# Short- and Long-Term Influences of Flipped Classroom Teaching in Physiology Course on Medical Students' Learning Effectiveness

**DOI:** 10.3389/fpubh.2022.835810

**Published:** 2022-03-28

**Authors:** Ming Ji, Ziqiang Luo, Dandan Feng, Yang Xiang, Jianping Xu

**Affiliations:** Department of Physiology, School of Basic Medical Science, Central South University, Changsha, China

**Keywords:** physiology, learning effectiveness, medical students, flipped classroom teaching, short- and long-term influences

## Abstract

The flipped classroom (FC) teaching has been increasingly employed in medical education. Many studies have shown this “student-centered” pedagogical model improves students' overall achievement in the course, with students showing more motivation and better self-directed learning skills when compared to the traditional classroom teaching. However, most of the previous studies have been evaluating the short-term effects of FC teaching conducted upon completion of the course. The retention of the promotion and the long-term effects on learning of students' subsequent courses deserve further attention and evaluation. By adopting and running FC teaching in the whole course of physiology, this study aimed to determine the short-term impact of FC teaching on students' learning of physiology course and also the long-term influences in students' learning of follow-up medical curriculums within 18 months after the completion of physiology course. 119 sophomore students majoring in clinical medicine from Central South University were recruited and they were assigned randomly into two groups: the control group (*n* =61) who received the traditional lecture (TL) teaching, and the experimental group (*n* =58), who received the FC teaching. In this study, students' final exam scores were used to assess their learning effectiveness and an independent samples *t*-test was conducted to compare scores between the two groups. Our study showed that FC teaching significantly improved the learning outcome of physiology in the experimental group compared with the control group (*P* = 0.0001) without obvious impact on the learning of other subjects conducted in the same period of time. Moreover, our results also demonstrated the long-term benefit of FC teaching, with students from the experimental group scoring higher in pathophysiology (*P* = 0.006), pathology (*P* = 0.029), pharmacology (*P* = 0.0089), diagnostics (*P* = 0.01) and internal medicine (*P* = 0.0004) than those from the control group. The study results indicate that FC is a promising teaching approach to increase students' learning effectiveness in physiology course, and the long-term effects of FC facilitate the learning of the follow-up medical courses. Furthermore, this study also demonstrates that although the time investment on physiology is increased by FC teaching, it does not weaken students' learning of other courses conducted in the same period of time.

## Introduction

With an increase in the amount of medical knowledge and the complexity of the health care system, it comes with great challenge for both the medical educators and the students, who have to juggle between increased curriculum content and reduced face-to-face teaching time. It is therefore necessary to make corresponding changes to the way we conduct medical education (1, 2). In recent years, the flipped classroom (FC) teaching method has been applied more often in medical undergraduate education. This teaching mode transfers the initiative of learning from teachers to students. Students are asked to do a basic learning through reading materials or videos before the face-to-face class, which is aimed to free up classroom time for knowledge application and higher-level thinking (2, 3). FC also provides students with sufficient time for discussion and interaction with their peers, which helps them develop critical and independent thinking and enhance their learning process (4–7). Many studies, either in preclinical or clinical settings, have demonstrated the advantages of FC over traditional classroom teaching, in improving students' course performance, and increasing their interest, participation and satisfaction with the course. So, this “student-centered” way of learning has been well-received by the students (7–9).

Learning effectiveness refers to any advancement in knowledge, skills, attitudes, and emotions after completing the course (10–12). Most of these studies have only investigated the short-term learning effectiveness in the course implemented with FC, by assessing the students' final examination results, students' participation and satisfaction with the curriculum, etc. A few studies looking at the long-term effects of FC, have however focused mainly on retention of the learned knowledge over a period of time, and their findings are rather inconsistent (10, 13–16). One important aspect of medical school training, besides retention of the learned knowledge, is to develop and enhance students' lifelong learning skills including clinical problem solving, and the ability to acquire new knowledge. Improvement of these lifelong learning skills would benefit the students in a longer term both in their study of other courses and later in their workplace (17, 18). Flipped classroom teaching, as a novel way of teaching, could potentially help students develop these lifelong learning skills by promoting the application of medical science knowledge, and stimulating critical thinking (1, 19, 20). Therefore, when evaluating the effects of FC teaching, we should include both the short-term and the long-term effects of FC by assessing students' learning effectiveness in both the current and the subsequent courses. However, there is currently a lack of reports on the long-term impact of FC. Our current study is aimed to explore the impact of FC on the learning outcomes of medical physiology and the long-term influences of FC on the learning of other courses conducted after the completion of medical physiology course. The choice of physiology course is because it provides the basic understanding on how our body functions under health and disease conditions, and it is known as “logic of life” (21). The understanding and mastery of physiological knowledge may affect the study of subsequent medical courses. In this study, we used learning effectiveness to reflect the influence of FC.

## Methods

### Design

We conducted this quasi-experimental study in a physiology course at Xiangya School of Medicine, Central South University. Most of the medical courses adopt the traditional lecture (TL) teaching method. Physiology course is conducted in the fourth semester. In 2016, we randomly selected two classes, one of which was subjected to TL teaching (the control group) and the other one was subjected to FC teaching (the experimental group). The test results were used to evaluate the physiology learning effectiveness of students in these two groups to assess the short-term benefit of FC teaching. An anonymous questionnaire survey among the students of experimental group was conducted to determine students' opinion regarding the FC teaching at the end of the course. At the same time, the final grades of medical microbiology, medical immunology and medical parasitology, which were conducted simultaneously in the same semester, were also analyzed and compared between these two groups to assess the impact of FC teaching on learning of these courses conducted using TL. The grades of major basic medical courses including pathophysiology, pharmacology, and pathology in the fifth semester, the final exam scores of clinical courses including diagnostics and internal medicine in the sixth semester and surgery pandect in the seventh semester were also compared between these two classes, to assess the long-term effectiveness of FC teaching.

### Settings and Participants

**Figure 1:**
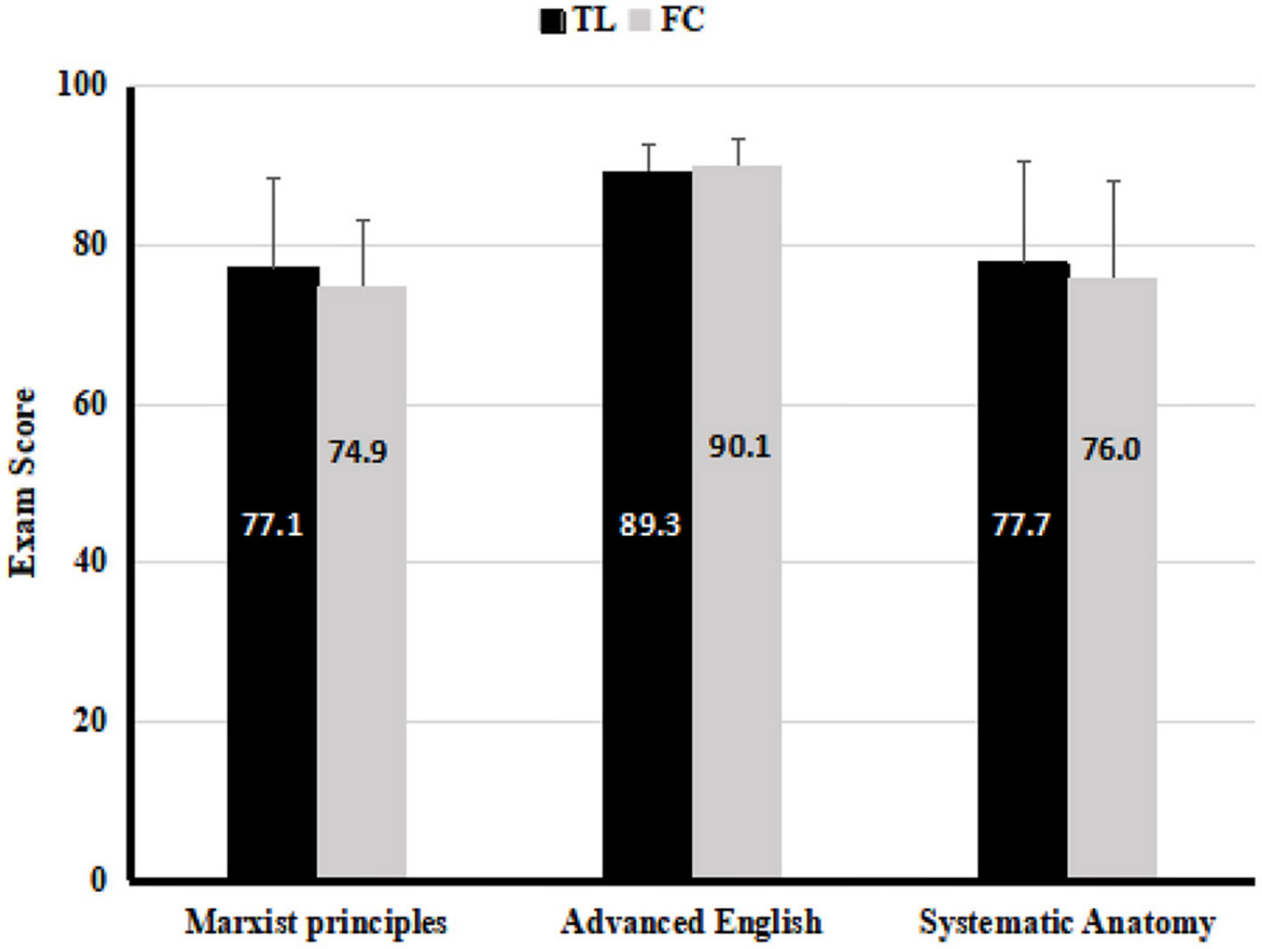
The average exam scores of previous courses in the TL teaching class (*n* = 61) and FC teaching class (*n* = 58), analyzed by *T*-test. Values are means ± SD.

In China, after passing the college entrance examination, senior high school students enter medical school for 5 years of undergraduate education in clinical medicine according to their applications. There are about 300 students enrolled in clinical medicine major and they are divided into 5 teaching classes every year. This study subjects were sophomores enrolled in 2014 with clinical medicine major. Students had been organized into 5 classes by the Office of Medical Educational administration, and each class had about 60 students. We randomly selected two classes, which were comparable in their final scores of Marxist principles, advanced English and systematic anatomy completed in the third semester. If there were statistical difference in the average scores of any of the three disciplines between two selected classes, a new random selection would be performed. Between the two classes we randomly selected at first, no significant differences in their grades of the above-mentioned principles were observed (Figure 1). One of the two selected classes received the TL teaching and was assigned as the control group (*n* = 61); the other one received FC teaching and was designated as the experimental group (*n* = 5 8).

### Intervention

These two classes were both taught by the same experienced teachers. The entire physiology teaching process of the experimental group adopted FC teaching. The experimental group established a QQ Group, for communication between students and teachers after class. The tasks and requirements of autonomous learning including video links were delivered to the students 3 days prior to the classroom teaching through the QQ Group. The reading materials for FC teaching in physiology come from iCourse (https://www.icourses.cn/home/), a learning platform established by the Higher Education Press of China. There are many shared resources for physiology from different medical universities in China on the iCourse platform, including one from Central South University created by ourselves (https://www.icourses.cn/sCourse/course_6701.html). Our physiology course resources include 72 classroom recorded videos, with durations between 36 to 56 min, [an average of 46.28 min per chapter (12 chapters in total)], 14 experimental teaching videos, 13 Chinese courseware and 12 English courseware (PDF version). These reading materials and videos cover all the teaching content of physiology, plus 12 sets of chapter test questions and 1 set of final test questions. Students can click the file and learn at their own pace. The experimental group students were required to follow the self-study guide by watching the instructional video on classroom learning, complete the exercises to consolidate the basic knowledge learned, write down the questions, and complete the preliminary transfer of knowledge. In the classroom, on the basis of explaining key points and difficult points incisively, a question-driven discussion teaching model was carried out, with interactive activities of “teacher asking questions - student discussion and answer - teacher induction and expansion.” Since the basic concepts and knowledge had been pre-acquired through autonomous learning before the classroom teaching, FC gave the teachers more time for in-depth explanation of the key and difficult “knowledge.” It also allowed the teacher to relate the knowledge to the history of discovery, to provide guidance for clinical application, and to answer the questions from the students submitted before class. During the interactive discussion session, teachers asked a series of questions which were vertically and horizontally expanded around 1–2 clinical cases. Students were divided into small groups (5–6 students/group) for discussion at the start of the class, and a representative from each group was selected to summarize and present the group's answer after the discussion. The teachers gave comments and elaborated further on the discussion and answers of the students. To ensure a balance between the discussion time and the lecturing time in the classroom, the discussion time for each teaching unit was controlled at about 30% of the total class duration. Throughout the course, three special clinical case studies with a total of 9 class hours, were arranged in stages to help students integrate and construct a complete physiology knowledge system. The choice of complex cases for group discussion (10–15 students/group) could encourage the integration of knowledge points in each system and complete the sublimation of “knowledge fragments” into the knowledge system. The control group, on the other hand, received the TL teaching, which was mainly in the form of lectures, without pre-class learning requirements and class discussions. Course content, number of classroom sessions and the duration of each class for the control group were the same as that of the experimental group. The control group also received three special clinical case studies, delivered in the same manner as for the experimental group. The control group also had a class QQ Group. Due to the difference in the course schedule, the communication between the two groups of students was limited. The physiology course lasted for 18 weeks. In week 20, students took the final closed-book examination. The exam paper consisted of a total of 100 points were prepared carefully by experienced teachers who did not take on teaching tasks. The types of questions included single-choice questions, short answer questions, and open-ended case analysis questions, accounting for 50, 20, and 30% of the total score respectively. The questions were all from Question Bank constructed by the department of physiology previously. The difficulty index and the discrimination index of the test paper was 0.58 and 0.41 respectively, and the reliability and the validity were 0.87 and 0.745 respectively.

### Data Collection

An online testing software program named AMEQP was used to review the physiology final exam papers. To eliminate the subjective deviation in the teacher's judgment, all subjective questions including short answer questions and open-ended case analysis questions, were marked by two independent blinded teachers, who did not know the name of the student, the identity of the other teacher and the grade given by the other teacher. The final score of each subjective question was the average of the scores from the two teachers. Grades of other courses were collected through the school educational administration achievement management system. Besides the exam scores, feedback from the experimental group students was also collected through an online anonymous questionnaire. The questionnaire was designed based on previous peers teaching researches in medical education (22, 23), consisting of five items of perceptions of active learning activities, academic engagement behavior, learning outcomes, interest, motivation, and aimed to assess students' perceptions of FC on learning and their attitudes toward FC on a Five-Point Rickett Scale. The responses include: strongly disagree, disagree, uncertain, agree and strongly agree. 58 questionnaires were received from the experimental group with a recovery rate of 100%.

### Data Analysis

To analyze learning outcomes, a post-test design was used to compare the final exam scores of the two groups of students. Courses included physiology and concurrently conducted (microbiology, immunology, and parasitology), follow-up basic medical courses (pathophysiology, pathology, and pharmacology) and main clinical courses (diagnostics, internal medicine, and surgery pandect). Exam scores were expressed as mean ± standard deviations. An independent sample *t*-test was used to compare mean grades between the control and the experimental groups, with a *P*-value < 0.05 considered statistically significant. Survey data for questionnaires were reported as percentages.

### Ethical Consideration

The study was reviewed and approved by the Ethics Committee of Central South University. Participants provided written informed consent to participate in the study.

## Results

### Baseline Information of Students

As shown in Figure 1, there were no significant differences between the two classes selected randomly in the scores of Marxist principles (*P* > 0.05), advanced English (*P* > 0.05) and systematic anatomy (*P* > 0.05). Based on this result, these two randomly selected two classes were eligible for this study. There were 61 students in the control group, of which 52% were women and 48% were men. The average age of students was 19.8 ± 0.72 years. The number of students in the experimental group was 58 with 49% females and 51% males, and the average age was 19.7 ± 0.69 years.

### FC Teaching Improves Students' Learning Effectiveness in Physiology Without Impacting Their Performance in the Other Courses Conducted in the Same Term

**Figure 2:**
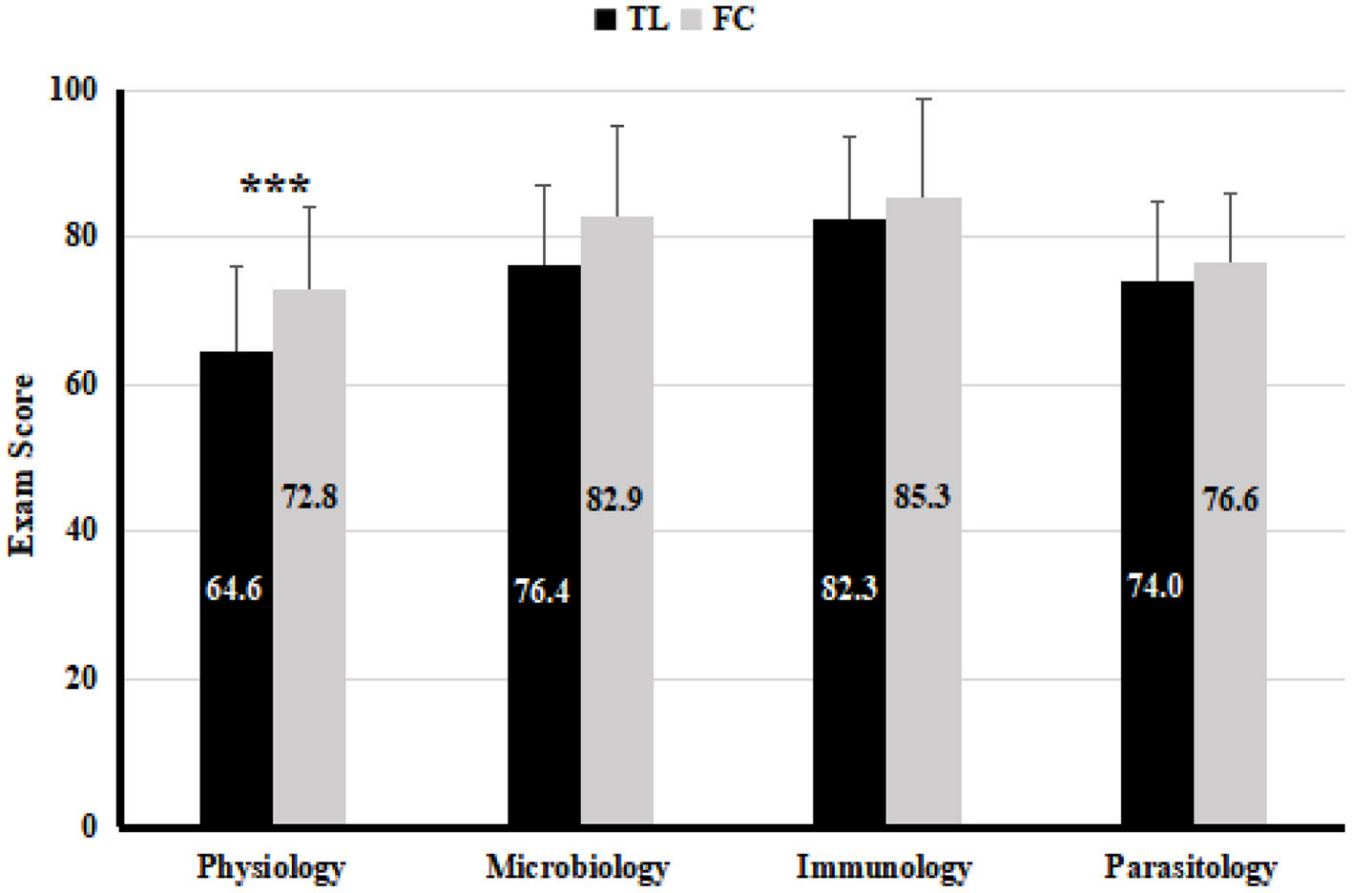
The average exam scores of physiology and the courses offered in the same period in the TL teaching class (*n* = 61) and FC teaching class (*n* = 58), analyzed by *T*-test. Values are means ± SD. ***P < 0.001.

To determine the short-term impact on the student's learning effectiveness, students' test scores of the physiology course were compared between the control and the experimental groups. Our results showed that the average score of the students is significantly higher in the experimental group than in the control group (*P* <0.001, see Figure 2), suggesting that the learning effectiveness was improved by the FC teaching. FC teaching required more preparation time from the students, we wanted to determine if this would impact the performance of students in the other 3 compulsory basic medical courses conducted in the same term. The final exam scores of the 3 courses carried out simultaneously with physiology were therefore compared between the control and the experimental groups. As shown in Figure 2, there were no significant differences between the two classes in the scores of microbiology (*P* > 0.05), immunology (*P* > 0.05), and parasitology (*P* > 0.05). Our result suggested that the learning of other courses in the same period was not significantly impacted by the FC teaching, which was consistent with previous study conducted in 2012 (24).

### FC Has Long-Term Positive Effects on Students' Learning Effectiveness

**Figure 3:**
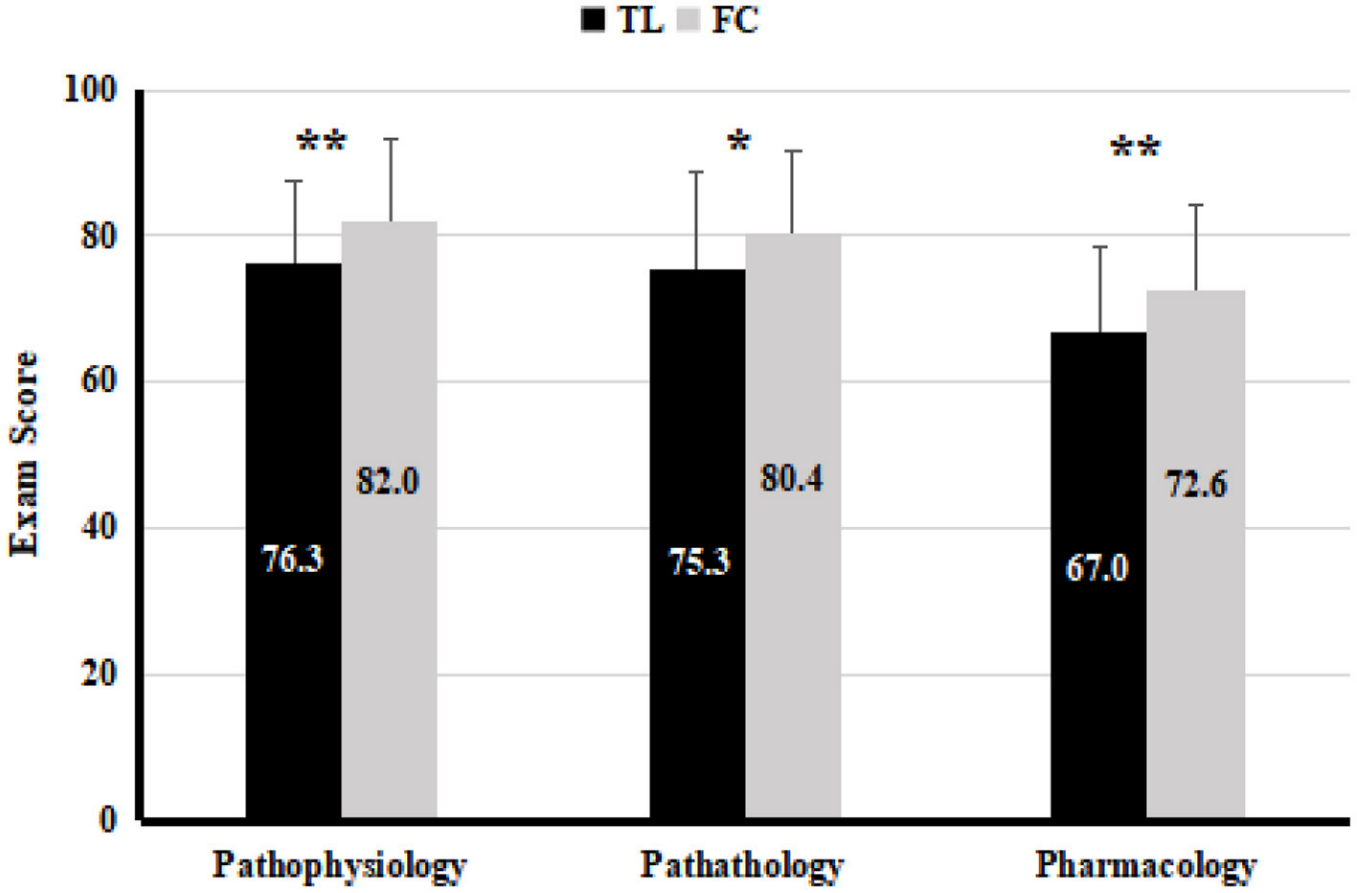
The average exam scores of follow-up basic medical subjects with physiology in the TL teaching class (*n* = 61) and FC teaching class (*n* = 58), analyzed by *T*-test. Values are means ± SD. *P < 0.05, **P < 0.01.

To determine whether the positive effects of FC as seen in the physiology course would continue to improve students' learning effectiveness in a longer term, the final exam scores of 3 basic medical courses (namely, pathophysiology, pathology, and pharmacology) conducted in semester 5, the following semester after completion of FC teaching, were compared between the control and the experimental groups. Our results showed that the scores of pathophysiology (*P* < 0.01), pathology (*P* < 0.05) and pharmacology (*P* < 0.01) were significantly higher in the experimental group than in the control group (Figure 3).

**Figure 4:**
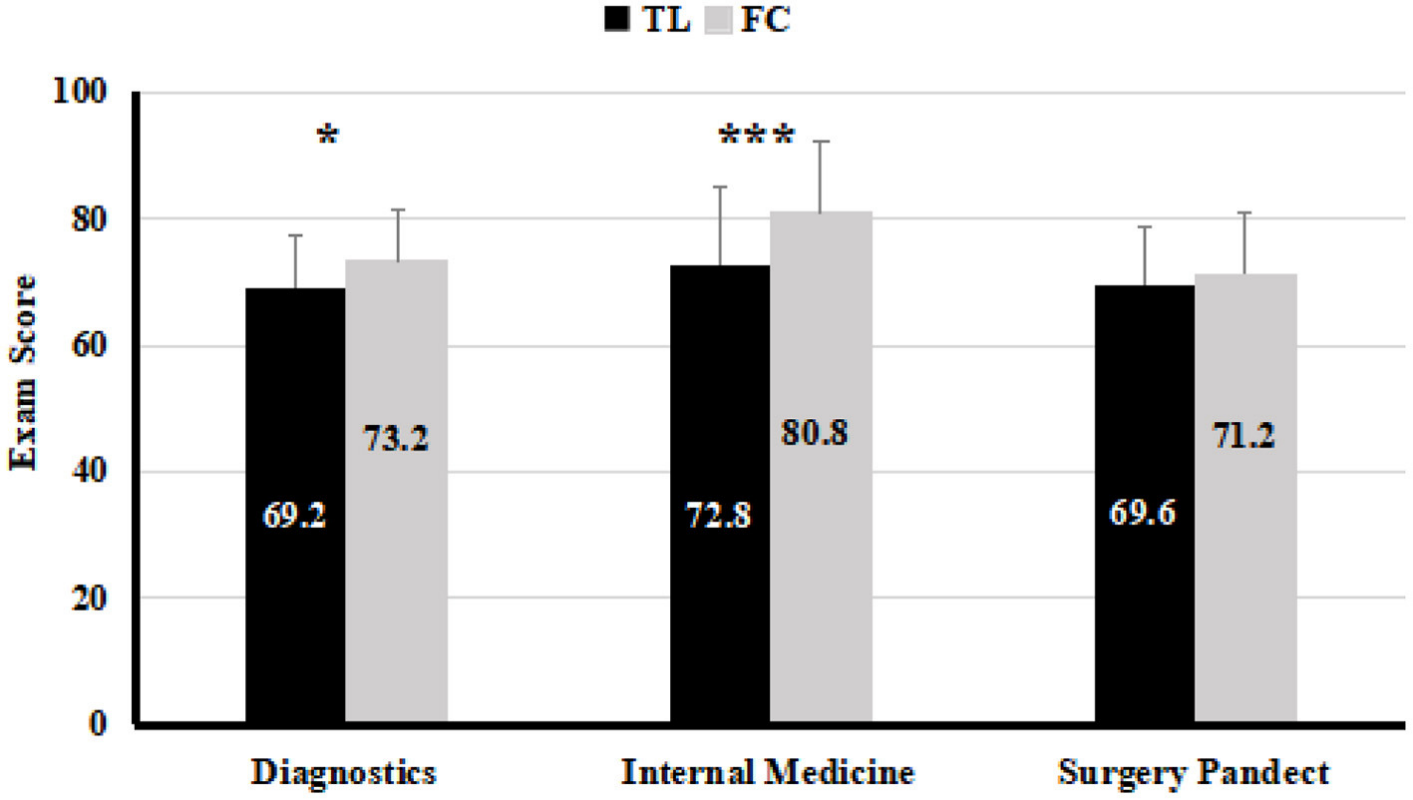
The average exam scores of clinical main courses in the TL teaching class (*n* = 61) and FC teaching class (*n* = 58), analyzed by *T*-test. Values are means ± SD. *P < 0.05, ***P < 0.001.

We continued tracking students' learning through semester 6 and 7 conducted. The final examination scores of three main clinical courses: diagnostics, internal medicine in semester 6 and surgery pandect in semester 7 were compared between the control and the experimental groups. As shown in Figure 4, the academic performance of diagnostics (*P* < 0.05) and internal medicine (*P* < 0.001) were significantly higher in the experimental group than in the control group. The scores for surgery pandect were however not significantly different between the two groups (*P* > 0.05). Our suggested that FC teaching had a long-term positive effect on the students' learning effectiveness in the courses conducted after the completion of FC teaching.

### Students' Views on Flipped Classroom Teaching

All students in the FC experimental group (*n* = 58) were invited to fill out a questionnaire, and all students (100%) responded. The results showed positive attitudes toward FC instruction with broadly agreed (response categories 3–5), ranging from 84.5 to 91.4% (see Table 1). About 89.6% of students thought that FC teaching enhanced logical thinking ability and about 86.2% of them thought that it also improved their critical thinking ability. Over 60% of students strongly agreed or agreed that they had learned a lot through this teaching model. 91.4% students acknowledged that they took more initiative in learning with the FC teaching model, and 87.9% thought it had improved their interest in learning. Nearly 90% of students were willing to recommend this course to other students.

**Table 1:** Students' perceptions of flipped classroom teaching in physiology (*n* = 58).

**Questions**	**Strongly agree**	**Agree**	**Uncertain**	**Disagree**	**Strongly disagree**
Discussions of questions in- class enhanced my logic ability	18 (31.0%)	20 (34.5%)	14 (24.1%)	3 (5.2%)	3 (5.2%)
It helped me to think critically	16 (27.6%)	20 (34.5%)	14 (24.1%)	4 (6.9%)	4 (6.9%)
I learned a lot taking this course	13 (22.4%)	24 (41.4%)	12 (20.7%)	4 (6.9%)	5 (8.6%)
Interactive, applied in-class activities enhanced my learning interesting	17 (29.3%)	18 (31.0%)	16 (27.6%)	5 (8.6%)	2 (3.5%)
It increased my motivation to study	26 (44.8%)	20 (34.5%)	7 (12.1%)	2 (3.5%)	3 (5.2%)
I was willing to recommend this course to another student	24 (41.4%)	18 (31.0%)	10 (17.2%)	3 (5.2%)	3 (5.2%)

## Discussion

Our research on FC teaching of physiology has demonstrated that FC can promote the learning effectiveness of physiology, and it continued to improve the learning effectiveness of the follow-up basic medical and clinical medical courses. Although FC teaching increased students' time investment during the course, it did not affect students' learning of the other courses conducted at the same time. The questionnaire survey showed that the FC teaching in physiology was well-received by the students who participated.

The strengths of FC include promotion of learning, consolidation of learning, unlimited opportunity for learning, and interactive learning (25). In the FC teaching, students learn factual basics through digital learning materials before class and can access online study materials without time or place restrictions (18, 25–27). Furthermore, free discussion with teachers and classmates during the class, gave the students opportunities to apply what they learned from self-study to problem-solving, and encouraged in-depth understanding of knowledge. FC teaching hence cultivates students' comprehensive ability and higher-order thinking skills for analyzing and solving complex problems (2, 25, 28). In a study conducted by Rianne A.M. Bouwmeester, on the second-year medical students who are taking Hematology and Oncology courses, FC teaching was also shown to allow more time for asking, answering questions, and discussion in class compared to the TL teaching. FC teaching saves the teachers time for explaining factual information and the time saved can then be used for explaining in-depth knowledge and responding to student questions (14). This teaching method is particularly suitable for basic science course such as physiology, which requires a deep understanding on the working mechanism of various body organs, the regulation mechanism of organ functions and is very useful for analyzing and solving clinical problems. Medical physiology, known as the logic of life, is a compulsory course for all medical students. It provides a theoretical basis for subsequent courses such as pathophysiology and pharmacology and is closely related to all other clinical subjects. Physiology is a discipline that requires high level of active and logical thinking. The learning content of physiology is abstract and logical, which is a challenging subject for students. In this study, we have shown that FC teaching improved the students' average test score in physiology by 12.7% compared to TL teaching. The findings from our study are consistent with previous studies which demonstrated improved performance of students by FC teaching in both preclinical and clinical courses (6, 18, 28–30). FC teaching has also been shown to help students develop and improve analytical thinking and problem-solving skills (28). Van Vliet's research demonstrates that the rate of correct answers to the exam questions, an indication of high-level cognitive thinking, is significantly higher in students receiving the FC teaching than those receiving the TL teaching (31). Chih-Cheng Lo's research found that flipped teaching enhanced students' motivation to learn and improved students' self-regulation ability (10). The results of the questionnaire in this study also showed the students' positive attitudes toward the FC teaching. The majority of the students agreed that FC teaching enhanced their logic and critical thinking skills. Students' interest in learning was greatly improved and their learning was more active. This study and the above researches suggest that FC teaching not only has a positive short-term promotion effect on learning effectiveness of this course, but more importantly, it enhances students' various learning abilities. Will the improvement of students' learning ability continue to promote students' performance in subsequent courses? Whether FC teaching can enable students to carry out high-level knowledge transformation and better performance, and whether it can enhance students' lifelong learning ability are questions yet to be answered (7, 9, 32). Therefore, it is necessary to follow and evaluate students' learning in their subsequent courses over a longer period of time after completion of FC teaching.

In our study, FC teaching was given to the experimental group in semester 4 and the learning effectiveness of students were followed and evaluated throughout semesters 5, 6, and 7. Except for one course, the surgery pandect, results of pathophysiology, pathology, pharmacology, diagnostics and internal medicine courses were better in students from the experimental group than those of the control group. This shows that the promotion effect of physiology FC teaching on students' learning effectiveness can remain to affect their subsequent course learning. Our study is among the few that are assessing the long-term impact of FC teaching on learning outcomes. Chih-Cheng Lo's research shows that students under the flipped teaching model make remarkable progress in the electronics course and the learning outcomes remains significant after a long period of time. The researcher believes that the FC teaching model can increase possibilities of long-term training or lifelong learning for students (10). A study showed that retention of knowledge in students taught by FC methodology were greater than those in the control group at 3 and 12 months after the completion of medical-surgical nursing course (12). However, findings on the knowledge retention were less consistent. Rianne A.M. Bouwmest's research has shown that students in FC have higher scores for self-efficacy. However, 10 months after the course, retention of knowledge and self-efficacy scores show no difference between control and FC teaching groups. The authors believed that although FC teaching did not prolong the retention time of knowledge, students receiving the FC teaching, were better in refreshing their knowledge when they were challenged to acquire knowledge on their own and were more proactive in class (14). It was also shown that the FC teaching was conducive for medical students to quickly adapt and complete tasks during the rotation of clinical departments (33, 34). This suggests that the positive effect of FC teaching on learning of subsequent course may not entirely be due to improvement of memory. FC teaching also helps promoting deep learning, strengthening the understanding and application of the acquired knowledge, and furthermore it trains students' logical, analytical and knowledge application skills. It is therefore not surprising that FC teaching is especially suitable for subjects which requires strong logical and active thinking skills, such as pathophysiology, pathology, pharmacology, as well as diagnostics and internal medicine (16, 35).

This study found that there was no significant difference in performance of surgery pandect between students receiving the FC teaching and those receiving TL teaching. This result is consistent with the findings by Whelan et al., which indicates that student-centered learning does not perform as well as those exposed to a more faculty-centered approach in anatomy laboratory exercises (36). FC teaching significantly improves students' ability to analyze material during final anatomical exams, but cognitive abilities related to memory is the same as that of traditional classroom teaching (37). This suggests that the effectiveness of the FC teaching on acquiring knowledge and skills at lower cognitive levels cannot be determined yet (9). It may also be that the content of the examination or assessment of surgery pandect and anatomy is related to a lower level of cognition and cannot comprehensively reflect changes in students' ability, or students taught in FC are likely to spend less time recalling and consolidating forgotten knowledge. All of the above requires further investigation.

The FC teaching requires students to spend a certain amount of time for self-study before each class, so it may increase the student's learning load (14, 26, 37, 38). Does this affect the study of other courses conducted simultaneously? To answer this question, the final exam results of subjects such as microbiology, immunology and parasitology that were carried out during the same period of time as the physiology were compared in our study. Our results found that FC teaching in physiology has no obvious impact on the learning of other courses conducted in the same period of time. Rianne A.M. Bouwmest's research has assessed this question from a different angle. Although students attending the FC spend about 2 more hours per week on homework than students in traditional education, they spent less time on the exam preparation compared to those receiving the TL teaching. The author believes that proper preparation and active participation in class will reduce the cramming learning before the exam (14). Future studies should therefore include the total time and time distribution among the different courses conducted in the same period. This will help determine the impact of the FC teaching on students' study-time management.

## Limitations of the Study

The limitation of this study is that exam scores were used as the sole indicator of the long-term impact of FC teaching on students' learning effectiveness. In addition, FC teaching was still at its early days back in 2016, so the curriculum resources were not comprehensive. At that time, the length of the classroom recorded video was too long, which meant more pre-class preparation time and increased workload for the students. We have since re-recorded 109 micro-lecture videos, each with a duration of 10–15 min, which give the students more time to manage other courses conducted at the same time. Secondly, the examination questions in our study were prepared by the teachers who did not undertake teaching, so there are no questions selected and designed which have been discussed in the FC, to assess the correct rate difference of answers between the two groups of students. Lastly, the number of research subjects enrolled in this study was relatively small, so interaction in the classroom was more effective. It would be interesting to see how FC teaching could be extended to all students, which requires more collective and consistent efforts from the teachers (28).

## Conclusions

The FC teaching in physiology has obvious positive influences on students' physiology learning effectiveness, and this effect can continue to improve the learning outcome of the follow-up basic medical and clinical medical courses. Furthermore, this study also demonstrates that although the FC teaching increases time investment for students during the course, it does not affect students' learning of other courses conducted in the same period of time. Therefore, the FC teaching can continuously influence the learning of other subjects and increase the possibility of lifelong learning. If FC were to be promoted on a larger scale, further studies would be required to assess students' compliance and find effective ways on motivating and guiding students during the pre-class preparation. Secondly, the training of teachers and the quality control of the teaching process are also the key issues to be considered.

## Data Availability Statement

The original contributions presented in the study are included in the article/supplementary material, further inquiries can be directed to the corresponding author.

## Ethics Statement

The studies involving human participants were reviewed and approved by Ethics Committee of Central South University. The patients/participants provided their written informed consent to participate in this study.

## Author Contributions

MJ and ZL conceived, designed research, and approved final version of manuscript. MJ, ZL, DF, YX, and JX performed experiments. MJ analyzed data, prepared figures, and drafted the manuscript. DF, YX, and JX interpreted results of experiments. MJ, ZL, and DF edited and revised manuscript. All authors contributed to the article and approved the submitted version.

## Funding

This work was supported by Central South University Education and Teaching Reform Fund Project (Nos. 2017jy79 and 2019jy142), the Research Fund for Curriculum Ideological and Political Construction of Universities in Hunan Province (hnkcsz-2020-0061), Research Project on Degree and Graduate Education Reform in Hunan Province (2020jgyb028), and Research project on curriculum ideological and political construction of Central South University (2020kcsz054).

## Conflict of Interest

The authors declare that the research was conducted in the absence of any commercial or financial relationships that could be construed as a potential conflict of interest.

## Publisher's Note

All claims expressed in this article are solely those of the authors and do not necessarily represent those of their affiliated organizations, or those of the publisher, the editors and the reviewers. Any product that may be evaluated in this article, or claim that may be made by its manufacturer, is not guaranteed or endorsed by the publisher.
